# Investigation of an international water polo tournament in Czechia as a potential source for early introduction of the SARS-CoV-2 Omicron variant into Belgium, Switzerland and Germany, November 2021

**DOI:** 10.2807/1560-7917.ES.2023.28.45.2300018

**Published:** 2023-11-09

**Authors:** Christoph Rudin, Nena Bollen, Samuel L Hong, Fanny Wegner, Lida Politi, Kassiani Mellou, Caspar Geenen, Sarah Gorissen, Bruno Verhasselt, Keith Durkin, Coralie Henin, Anne-Sophie Logist, Simon Dellicour, Tobias Resa, Tanja Stadler, Piet Maes, Lize Cuypers, Emmanuel André, Adrian Egli, Guy Baele

**Affiliations:** 1 University Children's Hospital Basel, Basel, Switzerland; 2 Department of Microbiology, Immunology and Transplantation, Rega Institute, KU Leuven, Leuven, Belgium; 3 Applied Microbiology Research, Department of Biomedicine, University of Basel, Basel, Switzerland; 4 Institute of Medical Microbiology, University of Zurich, Zurich, Switzerland; 5 European Programme for Intervention Epidemiology Training (EPIET), European Centre for Disease Prevention and Control, Stockholm, Sweden; 6 Department of Microbial Resistance and Infections in Health Care Settings, Directorate of Surveillance and Prevention of Infectious Diseases, Hellenic National Public Health Organization (EODY), Athens, Greece; 7 Directorate of Epidemiological Surveillance and Intervention for Infectious Diseases, Hellenic National Public Health Organization (EODY), Athens, Greece; 8 Department of Microbiology, Immunology and Transplantation, Laboratory of Clinical Microbiology, KU Leuven, Leuven, Belgium; 9 Department of Diagnostic Sciences, Ghent University Hospital, Ghent University, Ghent, Belgium; 10 Laboratory of Human Genetics, GIGA Research Institute, Liège, Belgium; 11 Federal testing platform COVID-19, Université libre de Bruxelles, Bruxelles, Belgium; 12 Spatial Epidemiology Lab (SpELL), Université Libre de Bruxelles, Bruxelles, Belgium; 13 Cantonal Office of Public Health Basel-Landschaft, Liestal, Switzerland; 14 Department of Biosystems Science and Engineering, ETH Zurich, Basel, Switzerland; 15 SIB Swiss Institute of Bioinformatics, Lausanne, Switzerland; 16 Department of Laboratory Medicine, National Reference Centre for Respiratory Pathogens, University Hospitals Leuven, Leuven, Belgium; 17 Clinical Bacteriology and Mycology, University Hospital Basel, Basel, Switzerland; 18 Swiss Pathogen Surveillance Platform (https://spsp.ch)

**Keywords:** COVID-19, SARS-CoV-2, Omicron, B.1.1.529, viral spread, phylogenetics, epidemiology, contact tracing

## Abstract

**Background:**

The earliest recognised infections by the SARS-CoV-2 Omicron variant (Pango lineage B.1.1.529) in Belgium and Switzerland suggested a connection to an international water polo tournament, held 12–14 November 2021 in Brno, Czechia.

**Aim:**

To study the arrival and subsequent spread of the Omicron variant in Belgium and Switzerland, and understand the overall importance of this international sporting event on the number of infections in the two countries.

**Methods:**

We performed intensive forward and backward contact tracing in both countries, supplemented by phylogenetic investigations using virus sequences of the suspected infection chain archived in public databases.

**Results:**

Through contact tracing, we identified two and one infected athletes of the Belgian and Swiss water polo teams, respectively, and subsequently also three athletes from Germany. In Belgium and Switzerland, four and three secondary infections, and three and one confirmed tertiary infections were identified. Phylogenetic investigation demonstrated that this sporting event played a role as the source of infection, but without a direct link with infections from South Africa and not as a superspreading event; the virus was found to already be circulating at that time in the countries involved.

**Conclusion:**

The SARS-CoV-2 Omicron variant started to circulate in Europe several weeks before its identification in South Africa on 24 November 2021. Accordingly, it can be assumed that travel restrictions are usually implemented too late to prevent the spread of newly detected SARS-CoV-2 variants to other regions. Phylogenetic analysis may modify the perception of an apparently clear result of intensive contact tracing.

**Key public health message**

**What did you want to address in this study?**

Immediately following its discovery in South Africa in late November 2021, the then new Omicron variant of SARS-CoV-2, was independently detected in Belgium and Switzerland in athletes returning from a water polo tournament in November 2021 in Czechia. We wanted to investigate, if this tournament could have served as a superspreading event and as the origin of the arrival and spread of Omicron in Belgium and Switzerland.

**What have we learnt from this study?**

We learned that Omicron had already been circulating in Europe when this sporting event occurred, weeks before its detection in South Africa. We also learned that, even when intensive contact tracing alone suggests a clear picture, namely a superspreading event at this tournament in our case, viral genome sequencing provides valuable additional insights that are essential for clarifying the real routes of infection.

**What are the implications of your findings for public health?**

When the SARS-CoV-2 Omicron variant was detected in South Africa in late November 2021, this immediately led to an almost complete stop of all international travel to and from South Africa. As shown by our investigation, travel bans are at best able to slow down but not likely to prevent the spread of newly detected SARS-CoV-2 variants to other regions, given today’s mobility.

## Introduction

Severe acute respiratory syndrome coronavirus 2 (SARS-CoV-2), the virus causing COVID-19, was first identified in Wuhan, China in December 2019 [1] and thereafter spread rapidly across the world. COVID-19 was declared a public health emergency of international concern (PHEIC) on 30 January 2020, and a global pandemic on 11 March 2020 by the World Health Organization (WHO) [2]. Efforts with substantial social, commercial, and economic consequences, such as lockdowns and international travel restrictions, have been undertaken worldwide to contain the spread of the virus. Despite these efforts and the rapid development of highly effective vaccines, the pandemic has resulted in more than 771 million recognised infections and more than 6.9 million deaths worldwide by 8 October 2023 [3].

**Figure 1:**
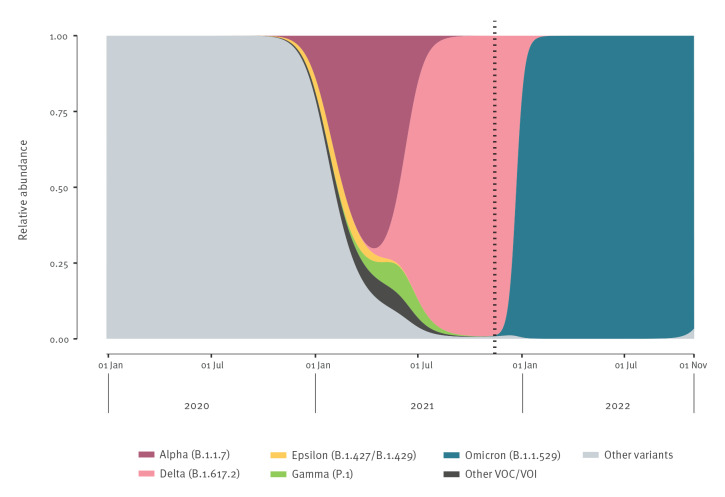
Lineage frequencies of SARS-CoV-2 over time since the start of the COVID-19 pandemic, focusing on the main variants of concern, worldwide, January 2020 up to October 2022

SARS-CoV-2: severe acute respiratory syndrome coronavirus 2; VOC: variant of concern; VOI: variant of interest.

Over the course of the pandemic, its epicentre shifted from Asia to Europe, South Africa, South America, and the United States, resulting in a series of pandemic waves driven by several viral variants of concern (VOCs, see Figure 1) [4-7], emerging because of immune escape and host adaptation [8]. The SARS-CoV-2 Omicron (Phylogenetic Assignment of Named Global Outbreak (Pango) lineage designation B.1.1.529) was reported by South Africa on 24 November 2021. Two days later, it was declared a VOC by the WHO. Omicron was recognised because of a rising number of SARS-CoV-2 samples with a spike gene target failure (SGTF) [9,10], caused by the presence of the 69–70 deletion in the spike (S) gene. Whole-genome sequencing (WGS) confirmed the presence of the new SARS-CoV-2 variant with a very large number of more than 30 amino acid changes in the spike protein [11]. Able to evade immunity against infection, Omicron started to rapidly spread globally, becoming dominant in Europe and most parts of the world until the end of the year 2021 (see Figure 1) [12]. The currently oldest sequenced Omicron sample was collected on 9 November 2021 in Botswana, according to GISAID [13]. The dynamics of its spread in South Africa and uncertainties about morbidity and mortality properties of this new variant led to an almost complete stop of all international travel from and to South Africa.

Pango lineages are listed after each variant name. Only individual VOCs that comprised over 5% of all sequences in a given week were included. The water polo tournament occurred at the very start of the wave of Omicron SARS-CoV-2 variant infections. The dotted vertical line indicates the timeframe of the water polo tournament (12–14 November 2021). Data for this figure were obtained from the GISAID public database [13].

Figure 1 illustrates the viral evolution and rapid worldwide distribution of emerging new variants of SARS-CoV-2. Large numbers of secondary infections caused by a single index case, referred to as ‘superspreading events’, have been recorded [14]. Superspreading events can affect institutions, such as assisted living/care facilities, and mass gatherings including sporting events [15-21]. Apart from general containment measures and a high vaccination rate, an effective targeted control of an epidemic relies on rapid discovery and isolation of infected individuals and identification of close contacts to quarantine them. In this regard, broad testing and contact tracing provide powerful instruments. ‘Backward’ tracing (tracing from whom disease spreads) is believed to be even more effective than ‘forward’ tracing (tracing to whom disease spreads) [22] and is especially helpful in identifying and handling superspreading events [23].

Here, we describe the results of an extensive investigation by means of contact tracing and molecular epidemiology, undertaken for better understanding of a suspected superspreading event – an international water polo tournament in Czechia – as a possible source of the arrival and early spread of the Omicron variant in Belgium, Switzerland and Germany in November 2021. 

## Methods

### Study context and setting

When the first SGTFs were independently detected in Belgium and Switzerland in November 2021, both countries suspected a connection to a possible superspreading event at an international water polo tournament, held between 12 and 14 November in Brno, Czechia. By means of contact tracing, both countries had come across female athletes infected with Omicron SARS-CoV-2 after having returned from this tournament. 

The Belgian team informed and consulted with the European Centre for Disease Prevention and Control (ECDC), which resulted in an Early Warning and Response System (EWRS) alert on 2 December 2021 and subsequently in collaboration of the Belgian and Swiss teams.

### Contact tracing

Forward and backward tracing were performed by phone and email by the first author (in Switzerland, and then Germany after having become aware of infections among German athletes from interviews with the infected Swiss athlete) and members of the Belgian tracing team at the University of Leuven. Apart from the infected individuals, the team coaches of the Belgian, German and South African water polo teams, and the team physician of the German team were contacted for details concerning travel, testing and (negative) results, accommodation, and contact with other teams.

### Sample collection and processing

Samples were collected in official local test centres and sent to accredited PCR laboratories. Samples with SGTF were then sent to national reference centres for WGS. 

### Molecular analyses

#### Minimum spanning tree analysis

To provide an initial impression of the similarity and relatedness between the collected samples, we constructed a minimum spanning tree (MST) as previously published [24]. We calculated a distance matrix with the alignment of Dataset C by (i) constructing an alignment of only polymorphic sites using SNP-sites 2.5.1 software [25] and (ii) computing a pairwise distance matrix on the alignment using Disty McMatrixFace v0.1.0 (https://github.com/c2-d2/disty). Following Walker et al. [24], we discarded n’s when computing the pairwise distances, and selected the 10 closest non-outbreak neighbouring sequences for each of the outbreak isolates, based on the distances previously computed. Finally, we performed a minimum spanning tree analysis for all outbreak sequences and their neighbours using the R package pegas v.1.2 [26] and visualised the resulting tree in Python using the networkX v2.6.3 [27] and matplotlib v3.3.4 [28] packages.

#### Phylogenetic analyses

##### Principles

Viruses mutate as they spread and the differences between virus genomes can be used to infer ancestral (or evolutionary) relationships among sampled individuals by means of phylogenetic inference. Genome sequences that are phylogenetically closer to one another are more likely to share an epidemiological association, which is the aim of the phylogenetic analyses carried out here (for more details on basic principles of phylogenetic analysis, see Supplementary Material S1).

##### Dataset composition

We performed three separate phylogenetic analyses with the different datasets. Dataset A includes sequences related to the water polo tournament from the three countries with contact tracing information (Belgium, Switzerland and Germany). Dataset B adds sequences from Czechia (where the sports event was held) and South Africa (presumed origin of Omicron) to Dataset A for a wider comparison. Dataset C corresponds to Dataset A including additional sequences from Belgium, Switzerland and Germany from up to 2 weeks after the final case linked to the water polo tournament (i.e. 4 weeks after the event) by contact tracing in order to find out if infections related to the tournament led to large transmission chains in the participating countries. For Datasets A and B, we downloaded all available SARS-CoV-2 genomes labelled as belonging to the B.1.1.529 (Omicron) strain from the GISAID database [13] that were sampled before 3 December 2021, from the aforementioned countries. For Dataset C, we used a cut-off date of 17 December 2021.

All collected samples were aligned using nextAlign and manually inspected for any inconsistencies (for details about quality checks, see Supplementary Material S2). After this step, we obtained the final dataset for each analysis. Dataset A included 360 sequences (Belgium, n = 53; Switzerland, n = 119; Germany, n = 188). Dataset B included 2,624 sequences (Belgium, n = 52; Switzerland, n = 118; Germany, n = 195; Czechia, n = 7; South Africa, n = 2,252). Dataset C included 3,822 sequences (Belgium, n = 1,049; Switzerland, n = 760; Germany, n = 2,013).

##### Methods of phylogenetic analyses

We performed maximum-likelihood (ML) phylogenetic inference using IQ-TREE v2.2.0 with automated model selection using the Bayesian information criterion (BIC) on each dataset. The use of such a model selection step is necessary to avoid overfitting and underfitting the data by means of the substitution model, which could lead to erroneous phylogenetic trees. We performed multiple replicates with increasingly demanding search settings and retained the phylogeny with the highest (log) likelihood. Specifically, we performed 10 replicates of each analysis with the default search settings of IQ-TREE v2.2.0 (only considering nearest neighbour interchanges in the vicinity of those previously applied, 100 initial parsimony trees, 20 top initial parsimony trees to optimise to initialise the candidate set, and maintaining 5 phylogenetic trees in the candidate set during ML tree searching), followed by another 10 replicates with the following settings: considering all nearest neighbour interchanges, 100 initial parsimony trees, 500 top initial parsimony trees to optimise and initialise the candidate set, and maintaining 75 phylogenetic trees in the candidate set during ML tree searching. We estimated a consensus tree using the ultrafast bootstrap (UFBoot) feature [29] in IQ-TREE v2.2.0, with 1,000 replicates. To time-calibrate the resulting ML phylogenies, we performed Bayesian inference through Markov chain Monte Carlo in BEAST 1.10 [30] on the fixed consensus topologies from the ML analyses, until sufficiently high effective sample size values (n > 200) were obtained, as assessed in Tracer v1.7 [31].

## Results

Independently of one another, our two research teams in Belgium and Switzerland found a possible link between the earliest Omicron infections and the water polo tournament that took place from 12 to 14 November 2021 in Brno in Czechia. The tournament hosted teams from Austria, Belgium, Czechia, Germany, South Africa, Switzerland and Wales. The Belgian team found two infected water polo athletes and four secondary infections, as well as three confirmed and three possible tertiary Omicron infections. The Swiss team recognised one infected athlete, three secondary infections and one tertiary infection. Furthermore, the Swiss team identified three infections in athletes on the German water polo team. An Early Warning and Response System (EWRS) alert of ECDC issued on 2 December 2021 initiated by the Belgian team resulted in a contact and close collaboration of the Belgian and the Swiss teams.

### Contact tracing

#### Switzerland

**Figure 2:**
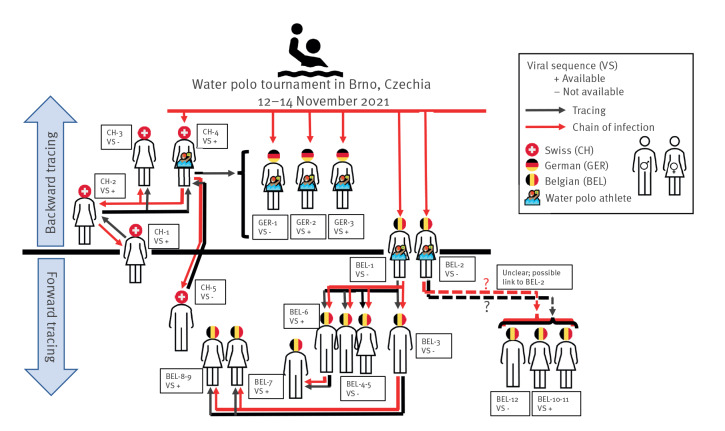
Contact tracing results among the SARS-CoV-2 Omicron cases related to the international water polo event, Belgium, Switzerland and Germany, 16 November–1 December 2021 (n = 20)

BEL: Belgium; CH: Switzerland; GER: Germany; SARS-CoV-2: severe acute respiratory syndrome coronavirus 2; VOC: variant of concern; WHO: World Health Organization.

**Figure 3:**
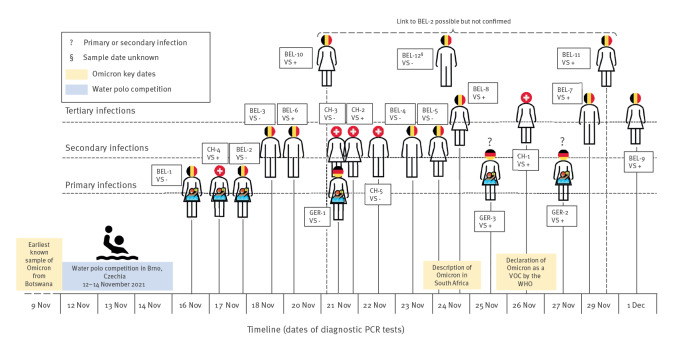
Timeline of detected primary (n = 6^a^), secondary (n = 7) and tertiary SARS-CoV-2 Omicron infections (confirmed n = 4; suspected n = 3) in Belgium, Switzerland and Germany, 16 November–1 December 2021

BEL: Belgium; CH: Switzerland; GER: Germany; SARS-CoV-2: severe acute respiratory syndrome coronavirus 2; VOC: variant of concern; WHO: World Health Organization.

^a ^Two of these cases could also be secondary infections (GER-2 and GER-3).

On 26 November 2021, a local laboratory reported a first SGTF in a SARS-CoV-2-positive PCR sample of a young nursery teacher (CH-1) in north-western Switzerland. Omicron was confirmed by WGS. Her sister (CH-2), living in the same household, was in isolation because of a COVID-19 infection at that time and turned out to be the only possible source of infection. Ten days earlier, on 16 November, this sister had ridden in a car with two of her colleagues to a swimming arena in the neighbourhood, without wearing masks. The driver of the car, a member of the Swiss national women’s water polo team (CH-4), became symptomatic and tested positive for SARS-CoV-2 by PCR on 17 November. The sister of the nursery teacher (CH-2) and the second passenger (CH-3) in the car were tested on 21 November; all three were confirmed to be infected with Omicron by PCR and sequencing. A complete sequence, however, was only available for two of them (CH-2 and CH-4). The water polo athlete (CH-4), working as a high-school teacher, was in contact with one of her students (CH-5) on 16 November, who also turned out to be infected with Omicron on 22 November (see Figures 2 and 3). 

Shown are all known Belgian (n = 12), Swiss (n = 5) and German (n = 3) infections related to the international water polo event of 12–14 November 2021 in Brno, Czechia, through a combination of backward and forward tracing.

These infections were found to be related to the international water polo event of 12–14 November 2021 in Brno, Czechia (symbols and abbreviations, see Figure 2).

#### Belgium

The first Belgian athlete (BEL-1) tested positive for Omicron on 16 November, followed by a positive PCR test for four close contacts (BEL-3 and BEL-4–6) between 18 and 24 November. Three tertiary SARS-CoV-2 infections were documented on 14 and 29 November and on 1 December (BEL-7 as close contact of BEL-6, and BEL-8–9 as close contacts of BEL-3). None of the close contacts of the second Omicron-positive Belgian athlete (BEL-2), diagnosed on 17 November, subsequently tested positive. Among more distant contacts, part of a family cluster of three infections (BEL-10–12) were identified, of which two were confirmed as Omicron by WGS; a link to BEL-2 was suggested but not conclusive according to contact tracing Figures 2 and 3.

#### Other countries

Apart from the affected Swiss and Belgian athletes, three members of the German team tested positive for SARS-CoV-2 infection after arrival home, with two confirmed as Omicron by WGS (GER-2 and GER-3; see Figures 2 and 3). No athletes from South Africa developed COVID-19-like symptoms during the tournament or after returning to South Africa.

### Water polo tournament

The Belgian and Swiss teams travelled to Brno from their countries via the Vienna airport in Austria by airplane and dedicated bus on 11 November, whereas the German team came from a training camp in Hungary. All members of the three teams tested negative by PCR at departure. On their way, they strictly adhered to the general applicable precautionary measures including physical distancing, hand hygiene and wearing masks. The members of the South African team were required to stay in self-isolation before leaving the country and required a negative PCR test before departure, as confirmed by the team coach. When arriving in Austria, they took a shuttle bus to Brno and all had another PCR test on arrival in the hotel.

In Brno, all teams resided in the same hotel and used the same facilities, including the common dining room and the bar. In principle, all members of the delegations were asked to wear face masks; however, they were not restricted in their social contacts.

During the tournament, Switzerland formed Group A together with Wales, South Africa, and Czechia, while Austria, Germany and Belgium formed Group B. Practice sessions were held on 11 November in groups: Belgium, Germany and Switzerland first, and Czechia, South Africa and Wales thereafter. On this day, an organising meeting was held in the hotel lobby, with the referee and one team representative from each country present.

Switzerland played twice against South Africa, first in the Group A match on 12 November and again in the final match on 14 November. Germany played against Belgium in the Group B match on 12 November, against South Africa in the semifinal on 13 November, and a second time against Belgium in the little final on 14 November. Before the day of the finals, there was an official event dinner at the hotel restaurant.

Finally, when departing the tournament on 14 November, the Swiss and German teams drove together from Brno to the airport of Vienna in the same bus. Although they were wearing masks, they were also sharing meals. The Belgian team returned home on 15 November. Before departure, the entire South African delegation took another PCR test, and all tests came back negative again. The South African referee was also part of the delegation and had to fulfil the same criteria.

### Molecular analyses

The Table shows an overview of the samples from individuals related to the international water polo tournament, along with their SARS-CoV-2 lineage assignments by Pango v.4.2 PANGO-v1.19 [32], as currently implemented in GISAID [33]. The current version of Pango distinguishes two different Omicron sublineages, BA.1 and BA.1.18, in our collected samples. The presence of more than a single Omicron sublineage most likely indicates the presence of at least two transmission clusters among our sampled individuals that were related to the international water polo tournament.

**Table:** SARS-CoV-2 Omicron Pango lineage assignments for the samples related to the international water polo tournament, Belgium, Switzerland and Germany, 16 November–1 December 2021 (n = 11)

Samples	Country	SARS-CoV-2 sublineage
BEL-6	Belgium	BA.1.18
BEL-7	Belgium	BA.1.18
BEL-8	Belgium	BA.1.18
BEL-9	Belgium	BA.1.18
BEL-10	Belgium	BA.1
BEL-11	Belgium	BA.1
CH-1	Switzerland	BA.1.18
CH-2	Switzerland	BA.1.18
CH-4	Switzerland	BA.1.18
GER-2	Germany	BA.1.18
GER-3	Germany	BA.1

BEL: Belgium; CH: Switzerland; GER: Germany; SARS-CoV-2: severe acute respiratory syndrome coronavirus 2.

Two Omicron Pango sublineages – BA.1 and BA.1.18 – were identified, leading to the assumption of multiple introductions and infection chains at the tournament.

**Figure 4:**
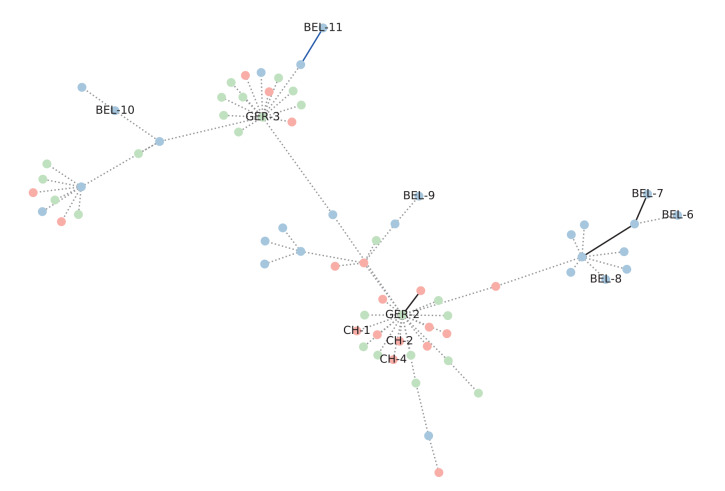
Minimum spanning tree between our samples of interest, 16 November–1 December 2021 (n = 11) and the 10 closest neighbouring sequences in Dataset C for each SARS-CoV-2 Omicron outbreak case, excluding the samples of interest, Belgium, Switzerland and Germany, 19 November–17 December 2021

BEL: Belgium; CH: Switzerland; GER: Germany.

To provide an initial impression of the potential clusters of infections, we constructed a minimum spanning tree (MST; Figure 4). The MST shows at least three clusters of viral strains circulating among our outbreak isolates. A cluster of Belgian sequences containing isolates BEL-6, BEL-7 and BEL-8 can be observed, with the remaining outbreak sequences scattered in clusters with a mix of sampling locations. These results suggest community spread in Belgium for one of the clusters and dispersal between Belgium, Switzerland and Germany for the remaining clusters. However, because the majority of edges in the MST have a distance of 0 and 1, the interpretation of partitioning the tree into clusters has to be done with caution, since multiple equally optimal minimum spanning trees can be obtained from a given distance matrix [34]. This uncertainty combined with the high genomic similarity between the isolates prevents us from using the MST to draw conclusions regarding the network structure of the transmission chain. Furthermore, an MST typically contains mostly outbreak sequences and few or no background sequences. 

Differences in branch lengths are only for clarity, as only the colour and style (dashed = 0, black solid = 1, blue solid = 2) refer to the number of nucleotide differences. Blue circles represent Belgian samples, red circles indicate Swiss samples and green circles show German samples. While there are only very few nucleotide differences between these samples, this specific MST suggests that three clusters are potentially present.

### Phylogenetic analyses

**Figure 5:**
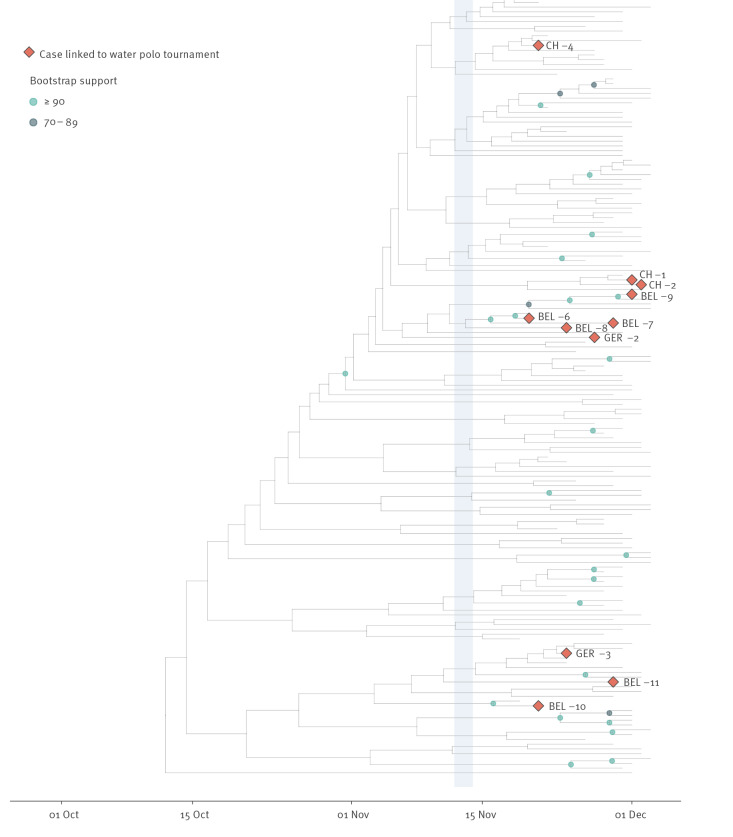
Phylogenetic analysis of the Belgian, Swiss and German SARS-CoV-2 Omicron cases related to the water polo tournament, 16 November–1 December 2021 (n = 11)

BEL: Belgium; CH: Switzerland; GER: Germany; SARS-CoV-2: severe acute respiratory syndrome coronavirus 2.

To carefully elucidate not only the evolutionary relationships between our collected samples, but also the role their infections play at the start of the SARS-CoV-2 Omicron wave of infections in Europe, we performed large-scale phylogenetic analyses. The key results of Analysis A (referring to Dataset A) are shown in Figure 5 (see Supplementary Figure S1 for the full phylogeny), revealing an estimated origin for the phylogeny in mid-August 2021 (16 August, 95% highest posterior density (HPD): 12 July–14 September), a few months before the water polo tournament. A first clade contains most of the infections related to the water polo event (CH-4, CH-1, CH-2, BEL-9, BEL-6, BEL-7, BEL-8, and GER-2) and has an estimated time to the most recent common ancestor (TMRCA) of early November 2021 (4 November, 95% HPD: 30 October–8 November), roughly 3 weeks before the first identification of the Omicron variant in South Africa. A second clade also has an estimated TMRCA of early November 2021 (3 November, 95% HPD: 23 October–13 November) and contains three infections possibly related to the international water polo event (BEL-10, BEL-11, GER-3). Of note, clustering with GER-3 may indicate a possible link of these infections to the water polo tournament, not evident by contact tracing. Interestingly, the Swiss sequences (especially CH-4) do not seem to cluster very close to the Belgian and German sequences. Further, the existence of these two clades, which each have a TMRCA older than that of the water polo tournament, may point to several separate transmission chains at the water polo tournament. In summary, a more widespread circulation of Omicron, rather than one single or even two source(s) of infection at the time of the event, is supported by the phylogenetic analysis.

The Swiss cases are shown to cluster in a subclade of a larger chain of infections that link most of the Belgian and German cases. Note that one of the Swiss genomes (CH-4) is distant from the other two. Only the part of the phylogeny relating the cases relevant to the water polo tournament is shown. The vertical blue shaded bar marks the time of the water polo tournament. The full phylogeny can be found in Supplementary Figure S1.

**Figure 6:**
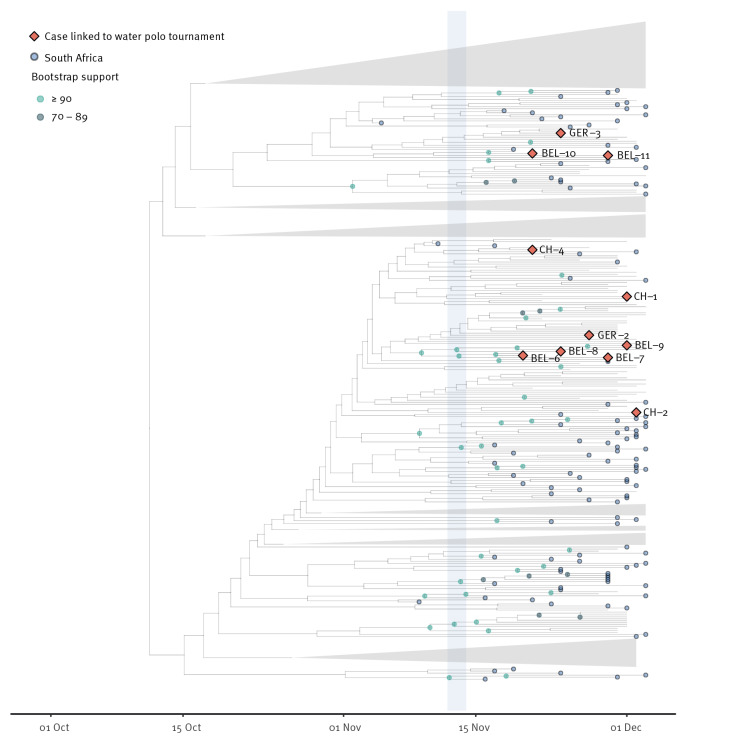
Phylogenetic analysis of the Belgian, Swiss and German SARS-CoV-2 Omicron cases related to the water polo tournament, 16 November–1 December 2021 (n = 11) against a background of genomes from Czechia and South Africa, 2 November–3 December 2021 (n = 2,259)

BEL: Belgium; CH: Switzerland; GER: Germany; SARS-CoV-2: severe acute respiratory syndrome coronavirus 2.

In Analysis B (Dataset B) we did not find evidence of clustering of South African sequences with those related to the water polo tournament (Figure 6). Given the vast number of Omicron genomes from South Africa during this time period (see Supplementary Figure S2 for the full phylogeny), it could be expected that the cases linked to the water polo tournament would be found in a South Africa-dominated cluster of infections. However, this was not the case, making it very unlikely that the South African delegation was a source of the infections among Belgian, Swiss and German athletes.

This phylogeny again shows two distinct clusters of infections, but the resolution among the Swiss genomes again proves difficult to resolve. The lack of intermingling of South African genomes among the relevant cases makes it very unlikely that South Africa was the origin of the infections stemming from the water polo tournament. Only the part of the phylogeny relating the cases relevant to the water polo tournament is shown. The vertical blue shaded bar marks the time of the water polo tournament. The full phylogeny can be found in Supplementary Figure S2.

**Figure 7:**
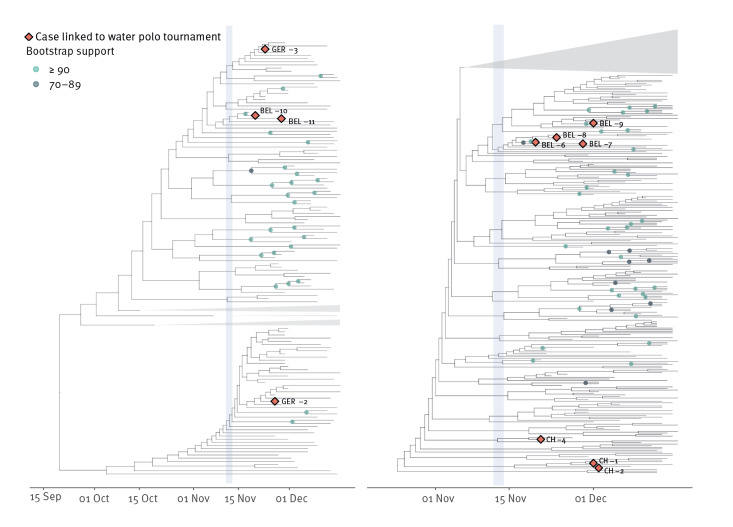
Phylogenetic analysis of the Belgian, Swiss and German SARS-CoV-2 Omicron cases related to the water polo tournament, 16 November–1 December 2021 (n = 11) with additional genomes from these countries, 17 November–17 December 2021 (n = 3,822)

BEL: Belgium; CH: Switzerland; GER: Germany; SARS-CoV-2: severe acute respiratory syndrome coronavirus 2.

Our final and largest Analysis C (Dataset C) assesses the impact of the events at the water polo tournament on the overall wave of Omicron infections in Belgium, Switzerland and Germany (see Figure 7). We investigated where the known cases are positioned within a large phylogeny as described above. We find that only few infections seem to stem from the documented cases and that no new clusters were sparked by any of the cases related to the water polo tournament. In other words, the water polo event did not immediately lead to large transmission chains of SARS-CoV-2 Omicron infections in Belgium, Switzerland, or Germany.

Genomes from an additional 2 weeks of genomic surveillance were included into this phylogenetic analysis. Because of the large number of genomes, two subclades were independently time calibrated. The phylogeny shows no evidence of large transmission chains originating from the infections at the water polo tournament. Only the aforementioned subclades of the phylogeny relating the cases relevant to the water polo tournament are shown. The vertical blue shaded bars mark the time of the water polo tournament. The full phylogeny can be found in Supplementary Figure S3.

## Discussion

Our report provides insight into a scenario of the initial dispersal dynamics of a new variant of SARS-CoV-2. The results of our contact tracing confirm the arrival of the Omicron variant in Europe by 14 November 2021 at the latest. However, initially we suspected a superspreading event at the water polo tournament in Brno with the South African team as the most likely source.

As demonstrated by Berggreen et al. using nine SARS-CoV-2 outbreaks in hospitals [35], WGS can be a valuable addition when it comes to clarifying an infection chain. In our case, phylogenetic analysis does not support our initial supposition based on contact tracing and points to an estimated TMRCA of these infections on 3 or 4 November, with the earliest uncertainty interval dating back to 23 October 2021. The combination of both methods provides evidence of an earlier spread of Omicron in Europe. This observation is consistent among our various phylogenetic analyses.

The identification of the Omicron variant on 24 November 2021 in South Africa and its declaration as a VOC by WHO on 26 November led to an immediate and almost complete stop of all international travel from and to South Africa. Even if new viruses or variants are identified rapidly, they are likely to be already circulating internationally when detected. Consequently, travel bans are likely to be implemented too late to completely prevent the spread of a new virus variant to other regions, as our example shows. This is especially true when viruses such as SARS-CoV-2 can be transmitted to others even before symptoms appear.

Despite our detailed contact tracing, we were not able to draw any conclusions about the exact manner of transmission or the exact time and location at which someone was infected during the water polo tournament. Transmission in the hotel, where all athletes resided, seems most likely; however, transmission between athletes could also have occurred in the swimming arena. The bus ride from Brno to Vienna could also have been a source of transmission between the German and Swiss athletes. Despite combining our phylogenetic and contact tracing results, we do not know how these infections came to Brno and if all individuals under investigation really acquired their infection at the tournament. We are certain however, that some athletes who had previously tested negative for SARS-CoV-19, arrived home from Brno with a SARS-CoV-2 Omicron infection. In any case, this tournament did not represent a superspreading event that resulted in large transmission chains in the countries involved.

Our study had some limitations. Unfortunately, some of the samples of the people we studied were not stored and therefore not available for WGS. Their corresponding genomic sequences are therefore absent from our phylogenetic analyses. However, the slight variations between the different analyses mentioned above are not surprising, as large-scale phylogenetic reconstructions (as in our analyses of Datasets B and C) of SARS-CoV-2 genomes are known to be very challenging due to the large number of sequences in conjunction with the low number of mutations [36]. Furthermore, the other countries that had delegations attending the water polo tournament did not report any cases associated with this sports event, and hence we have included – to the best of our knowledge – all relevant genomic information into our phylogenetic analyses. Finally, we are unable to dismiss the possibility that cases related to the sports event remained unknown but still entered publicly available databases. However, given the huge number of available genomes and the limited differences between those genomes, such cases are next to impossible to uncover through phylogenetic analysis alone.

## Conclusions

Our report demonstrates a time gap between the arrival of the Omicron variant of SARS-CoV-2 in several European countries and its identification in South Africa. Thus, even if a travel ban might slow down the spread, it is unlikely to prevent the introduction of a new variant to other countries/regions of the world. Furthermore, our report demonstrates how supplementing contact tracing with a phylogenetic investigation may add insights into suspected infection chains and modify the perception of an apparently clear result of intensive contact tracing.

## Supplementary Data

340000 Supplement
