# Supplementary material for: Investigation of an international water polo tournament in Czechia as a potential source for early introduction of the SARS-CoV-2 Omicron variant into Belgium, Switzerland and Germany, November 2021
Source: Euro Surveill. 2023 Nov 9;28(45):2300018. doi: 10.2807/1560-7917.ES.2023.28.45.2300018 (PMC10636743; doi:10.2807/1560-7917.ES.2023.28.45.2300018)

Supplement

Disclaimer

This supplementary material is hosted by Eurosurveillance as supporting information alongside the article '**Investigation of an international water polo tournament in Czechia as a potential source for early introduction of the SARS-CoV-2 Omicron variant into Belgium, Switzerland and Germany, November 2021'** on behalf of the authors who remain responsible for the accuracy and appropriateness of the content. The same standards for ethics, copyright, attributions and permissions as for the article apply. Eurosurveillance is not responsible for the maintenance of any links or email addresses provided therein.

Supplementary Material S1

Detail of principles of phylogenetic analysis

The combination of virus genomes and their associated sampling times can be used to inform a molecular clock, enabling the inference of time-stamped phylogenetic trees. In practice, maximum-likelihood inference is used to infer the most likely hypothesis about the evolutionary history that links the available samples, owing to its computational efficiency for large genomic datasets. Given that all large-scale inference methodologies employ heuristic approaches, we performed multiple replicates of each analysis – and selected the optimal one – to ensure an as accurate as possible phylogenetic result.

Supplementary Material S2

Detail of dataset quality check

We checked for the presence of temporal signal and for quality issues in the dataset by performing root-to-tip regression analysis in TempEst [1] using a maximum likelihood (ML) phylogeny obtained through IQ-TREE v2.2.0 with automated model selection [2]. Any sequences which were considered outliers in TempEst were removed.

---

[1] Rambaut A, Lam TT, Carvalho LM, Pybus OG. Exploring the temporal structure of heterochronous sequences using TempEst (formerly Path-O-Gen. Virus Evolution 2016 ; 2 :1-7
[2] Minh BQ, Schmidt HA, Chernomor O, Schrempf D, Woodhams MD, von Haeseler A, Lanfear R. IQ-TREE 2: New Models and Efficient Methods for Phylogenetic Inference in the Genomic Era. *Molecular Biology and Evolution 2020 ;* 37 :1530–1534

Supplementary Figure S1

Complete time-calibrated phylogeny relating all Belgian, Swiss and German Omicron genomes available on GISAID until 3 December 2021.

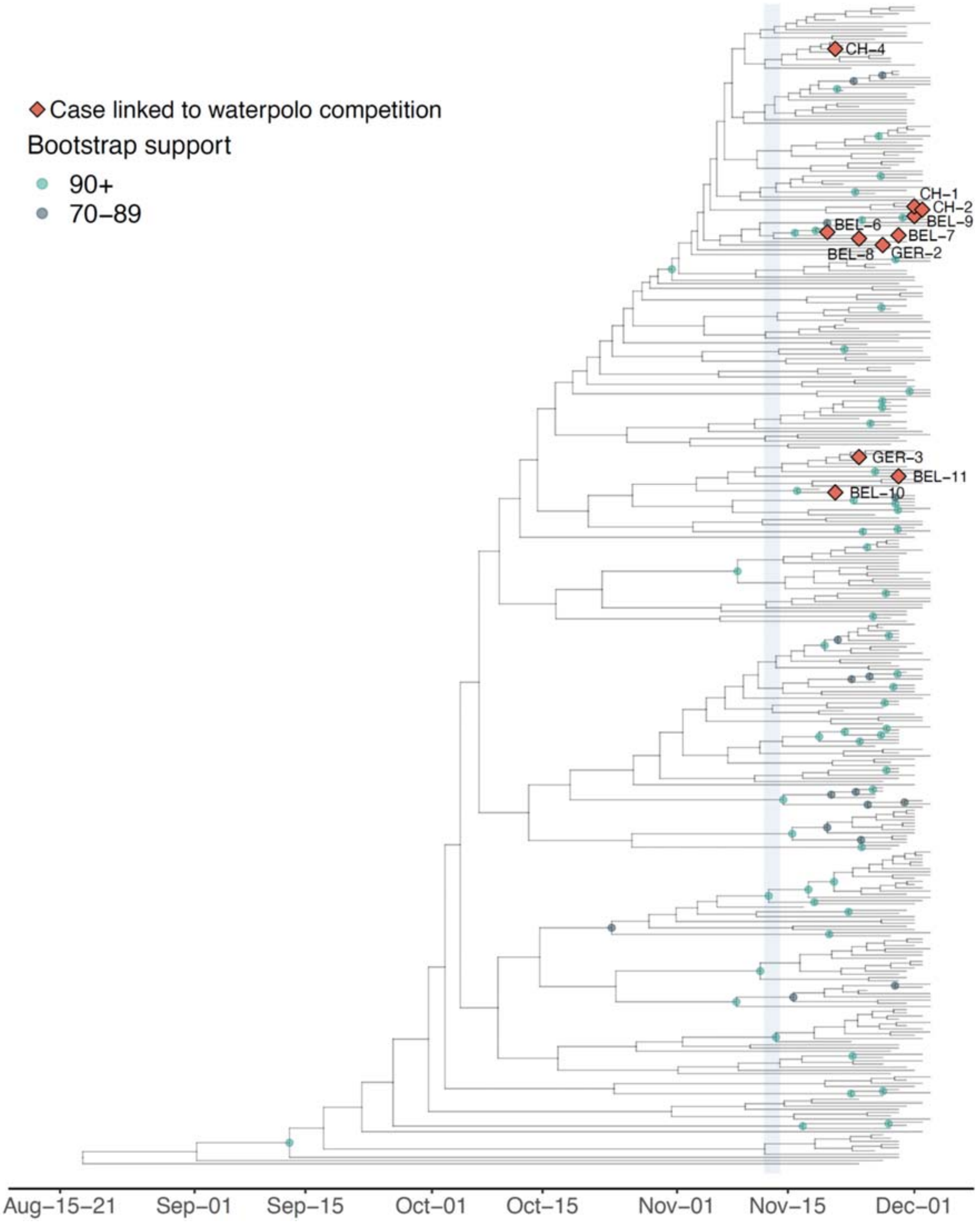

Supplementary Figure S2

Complete time-calibrated phylogeny relating all Belgian, Swiss, German, Czech and South African Omicron genomes available on GISAID until 3 December 2021.

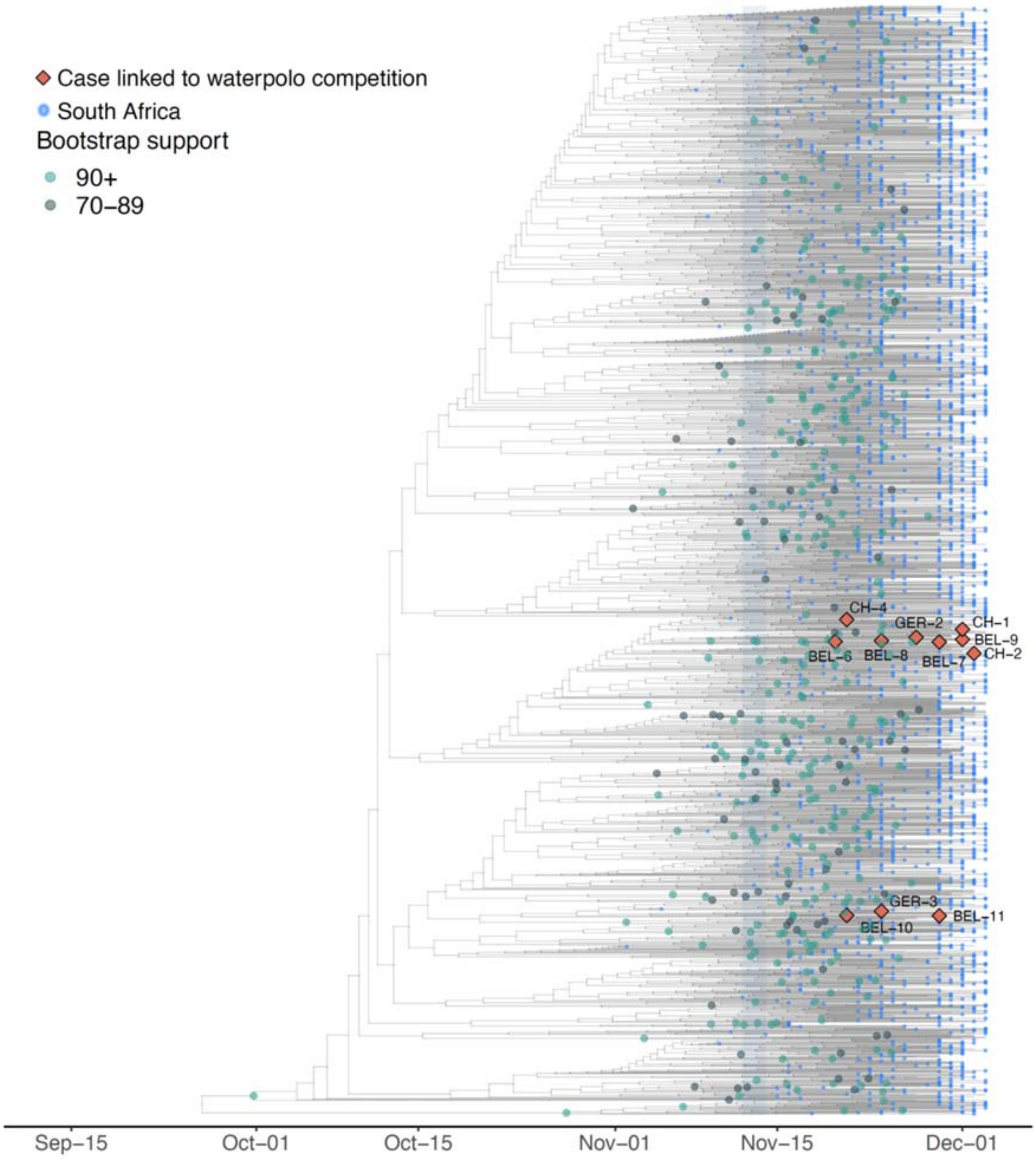

Supplementary Figure S3

Complete unrooted phylogeny relating all Belgian, Swiss and German Omicron genomes available on GISAID until 17 December 2021.

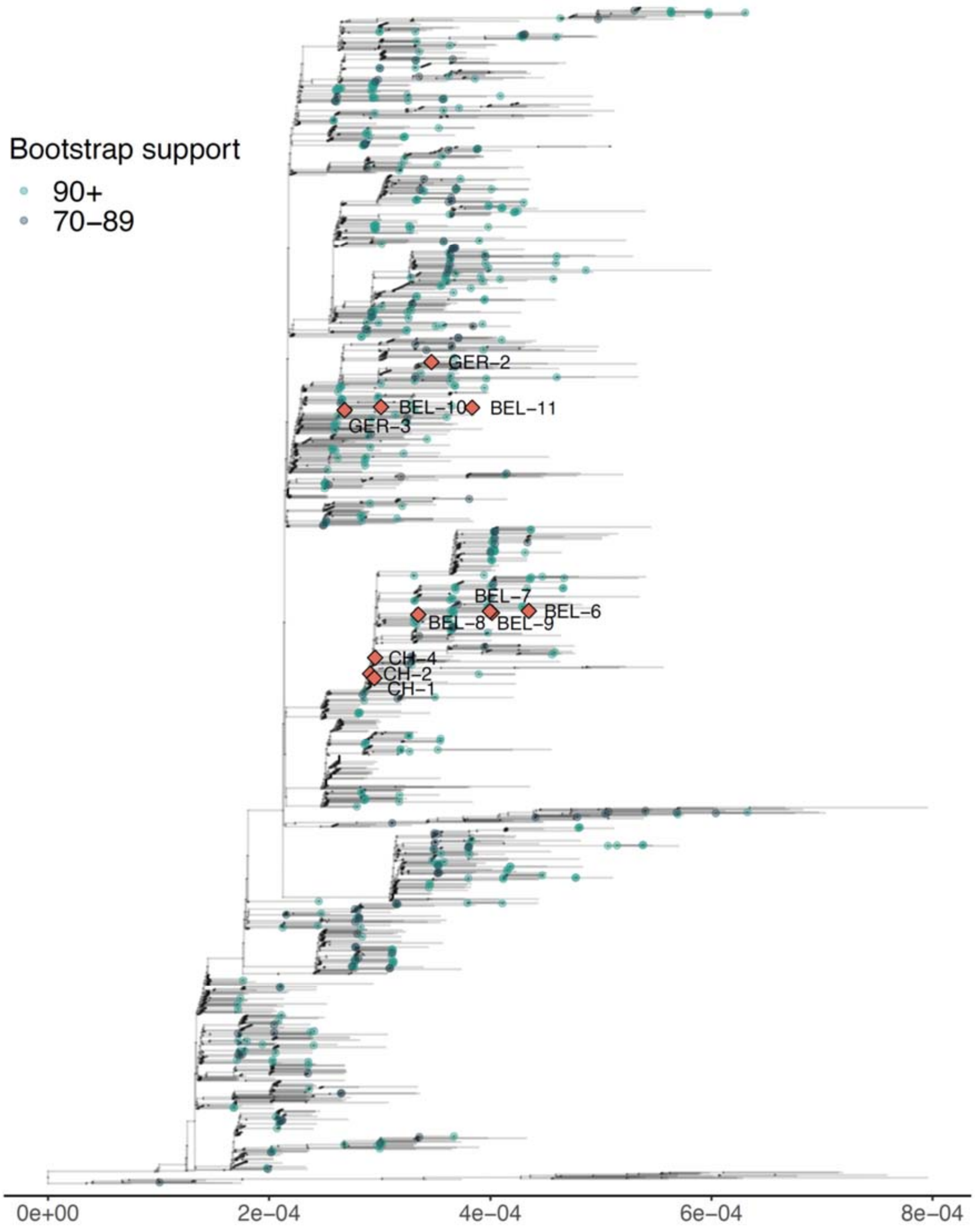

Supplement: Supplement [file 23-00018_RUDIN_Supplement.pdf]
